# Structural Comparison of the SARS CoV 2 Spike Protein Relative to Other Human-Infecting Coronaviruses

**DOI:** 10.3389/fmed.2020.594439

**Published:** 2021-01-14

**Authors:** Marni E. Cueno, Kenichi Imai

**Affiliations:** Department of Microbiology, Nihon University School of Dentistry, Tokyo, Japan

**Keywords:** C-terminal domain (CTD), modeling, N-terminal domain (NTD), SARS coronavirus 2 (SARS2), spike

## Abstract

Coronaviruses (CoV) are enveloped positive-stranded RNA viruses and, historically, there are seven known human-infecting CoVs with varying degrees of virulence. CoV attachment to the host is the first step of viral pathogenesis and mainly relies on the spike glycoprotein located on the viral surface. Among the human-infecting CoVs, only the infection of SARS CoV 2 (SARS2) among humans resulted to a pandemic which would suggest that the protein structural conformation of SARS2 spike protein is distinct as compared to other human-infecting CoVs. Surprisingly, the possible differences and similarities in the protein structural conformation between the various human-infecting CoV spike proteins have not been fully elucidated. In this study, we utilized a computational approach to generate models and analyze the seven human-infecting CoV spike proteins, namely: HCoV 229E, HCoV OC43, HCoV NL63, HCoV HKU1, SARS CoV, MERS CoV, and SARS2. Model quality assessment of all CoV models generated, structural superimposition of the whole protein model and selected S1 domains (S1-CTD and S1-NTD), and structural comparison based on RMSD values, Tm scores, and contact mapping were all performed. We found that the structural orientation of S1-CTD is a potential structural feature associated to both the CoV phylogenetic cluster and lineage. Moreover, we observed that spike models in the same phylogenetic cluster or lineage could potentially have similar protein structure. Additionally, we established that there are potentially three distinct S1-CTD orientation (Pattern I, Pattern II, Pattern III) among the human-infecting CoVs. Furthermore, we postulate that human-infecting CoVs in the same phylogenetic cluster may have similar S1-CTD and S1-NTD structural orientation. Taken together, we propose that the SARS2 spike S1-CTD follows a Pattern III orientation which has a higher degree of similarity with SARS1 and some degree of similarity with both OC43 and HKU1 which coincidentally are in the same phylogenetic cluster and lineage, whereas, the SARS2 spike S1-NTD has some degree of similarity among human-infecting CoVs that are either in the same phylogenetic cluster or lineage.

## Introduction

Coronaviruses (CoV) are enveloped positive-stranded RNA viruses that belong to the family *Coronaviridae* and order *Nidovirales* with the subfamily *Othocoronavirinae* composed of four genera, namely: *alphacoronavirus, betacoronavirus, gammacoronavirus*, and *deltacoronavirus* (1). Historically, there are seven known CoVs capable of infecting humans, namely: human CoV (HCoV)-229E (1962), HCoV-OC43 (1967), severe acute respiratory syndrome (SARS)-CoV 1 (SARS1) (2002), HCoV-NL63 (2004), HCoV-HKU1 (2005), Middle East respiratory syndrome (MERS)-CoV (2012), and SARS-CoV 2 (SARS2) (2019) (2–8). In general, CoVs cause serious health problems to both human and animal hosts and, in particular, CoV infections primarily affect the respiratory and gastrointestinal tracts (9). Moreover, CoVs have the largest genome among all known RNA viruses which in-turn is packed in a helical capsid comprised of a nucleocapsid protein (N) and surrounded by a viral envelope which in-turn is associated with structural proteins, namely: membrane, envelope, and spike (10). Among the structural proteins, the spike protein has been involved in mediating viral entry, determinant of host tropism, inducing viral pathogenesis, and major inducer of host immune responses (9–12). This would highlight the significance of the CoV spike protein in terms of viral pathogenesis.

Spike protein (a class I viral fusion protein) follows a metastable prefusion conformation upon translation and, likewise, forms trimers that resemble club-shaped spikes along the CoV membrane surface (13). Additionally, the spike protein is comprised of three segments, namely: the large ectodomain, single-pass transmembrane anchor, and short intracellular tail (10). With regards to the ectodomain, it is further divided into the S1 receptor-binding subunit that mainly functions in viral attachment and S2 membrane-fusion subunit that facilitates virus-cell fusion (9, 10). In a CoV infection scenario, S1 would bind to a suitable receptor on the host cell surface enabling viral attachment and, subsequently, S2 fuses both the host and viral membranes, thereby, allowing viral genomes to enter host cells (9, 10). This shows that receptor binding and membrane fusion are important initial and key steps in CoV pathogenesis. Interestingly, the receptor-binding domain (RBD) and host receptor differ among the known human-infecting CoVs. In particular, known host receptors include: aminopeptidase N (APN) for 229E; angiotensin-converting enzyme 2 (ACE2) for NL63, SARS1, and SARS2; *O*-acetylated sialic acid (*O*-ac Sia) for OC43 and HKU1; and dipeptidyl peptidase-4 (DPP4) for MERS (9, 14). Among the human-infecting CoVs, infection in the upper respiratory tract has been associated with 229E, OC43, NL63, and HKU1 (15), whereas, infection in the lower respiratory tract has been associated with SARS1, MERS, and SARS2 (7, 16, 17). Interestingly, among the human-infecting CoVs, only SARS2 infection resulted to a pandemic which would insinuate that the protein structure of the SARS2 spike protein has a structural conformation that is distinct as compared to other human-infecting CoVs (18, 19). However, to our knowledge, the structural comparison between SARS2 and the other human-infecting CoVs has not been fully elucidated. A better understanding of the possible differences and similarities in the protein structural conformation of the SARS2 spike protein compared to the spike proteins of the other human-infecting CoV may shed a light on how this particular virus more effectively cause infection and, more importantly, establish the potential cross-reactivity of SARS2 with other human-infecting CoV which in-turn may lead to novel therapeutic strategies.

## Materials and Methods

### CoV Spike Modeling

Representative CoV spike amino acid sequences from 229E, OC43, NL63, HUK1, SARS1, MERS, and SARS2 were collected from the National Center for Biological Information (NCBI) and UniProt Web sites. To obtain the most accurate monomeric spike model that could serve as the representative prefusion model for each CoV strain, a minimum of five generated sequence models were first analyzed and spike models with similar Root Mean Square Deviation (RMSD) values and Template Modeling scores (Tm-scores) based on superimposition using the default setting of Tm align (20) were used for further downstream analyses. The following representative amino acid sequences were utilized for spike modeling with Genebank accession number indicated: 229E (ABB90513), OC43 (AXX83297), NL63 (QED88040), HKU1 (ARB07617), SARS1 (AAR07625), MERS (AHX00731), and SARS2 (YP_009724390). In addition, representative SARS2 spike S1 C-terminal domain (S1-CTD) and N-terminal domain (S1-NTD) models were generated based on UniProt reference number P0DTC2. In the whole study, all generated models were made using the default settings of Phyre2 web server (21) and Jmol applet (22) was used for protein visualization.

### Model Quality Assessment

All generated spike models were assessed for quality prior to further analyses. Both protein model:crystal structure superimposition and contact mapping were utilized for model quality estimation. Representative crystal structure used for superimposition was the 1998 strain (PDB ID: 6VXX). Additionally, a monomeric 6VXX model (crystal model) was generated using Phyre 2 and, subsequently, superimposed for comparison to the 6VXX crystal structure to further serve as model quality check. Representative CoV spike models and crystal structure were superimposed using Tm align (20). For the purpose of this study, we considered spike models adequate for further analyses if RMSD values between superimposed sequence model:crystal and crystal model:crystal are close. Moreover, CMView applet (Contact type: Cα; Distance cut-off: 8.0; Needleman-Wunsch alignment) was used to establish protein contact map of both the model and crystal in order to determine common contact (23) and, consequently, higher common contact would mean more structural similarities between the model and crystal (24) which in-turn would further indicate whether the model is suitable for further analyses.

### Comparison Among CoV Spike Models

Three different sets of structural comparisons were performed. In one analysis conducted, all generated CoV spike models were compared (Visually, RMSD value, and Tm score) to the SARS2 spike model through superimposition. Subsequently, superimposition and comparison (both RMSD value and Tm score) between the various CoV spike models were likewise made. In another separate analysis, SARS2 spike S1-CTD and S1-NTD models were similarly superimposed and compared (Tm score only) to the other generated CoV spike models. For mutant spike model comparisons, superimpositions were done with the following: (1) original SARS2 spike model; and (2) original SARS2 spike S1-CTD and S1-NTD models. Visual observation (simply looking at the structure), RMSD value, Tm score, and protein common contact were established using Jmol, Tm align, and CMView, respectively.

## Results

### Generated CoV Spike Models Are Reliable

**Figure 1:**
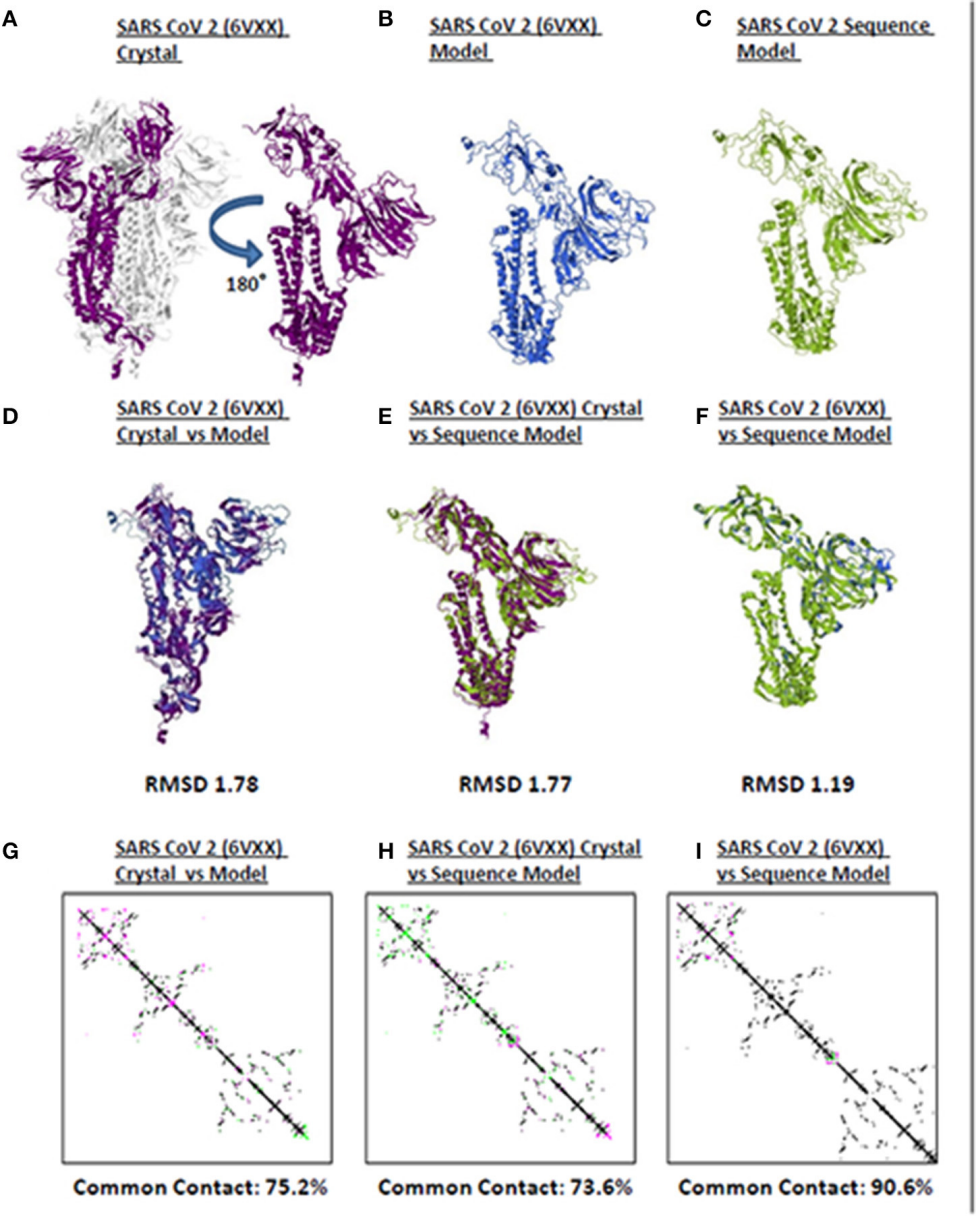
Quality assessment of monomeric human-infecting coronavirus spike protein models generated. Representative SARS CoV 2 **(A)** 6VXX crystal, **(B)** 6VXX model, and **(C)** sequence model of monomeric spike proteins are shown. Sumperimposition between **(D)** 6VXX crystal and model, **(E)** 6VXX crystal and sequence model, and **(F)** 6VXX and sequence models are presented. RMSD scores of the superimposed protein structures are indicated below. Contact maps of **(G)** 6VXX crystal and model, **(H)** 6VXX crystal and sequence model, and **(I)** 6VXX and sequence models are shown. Contacts present in both protein structures (black) and present in one of the protein structures [either pink (first protein structure uploaded) or green (second protein structure uploaded)] are indicated. Common contact of the protein structures being compared are labeled below. SARS CoV 2 6VXX crystal (violet), 6VXX model (royal blue), and sequence model (yellow green) are indicated.

Model quality assessment prior to further downstream analyses on either experimental (i.e., crystallized) or theoretical (i.e., computer-based) protein structures generated has long been recommended (25). In line with this, to elucidate the accuracy and reliability of all CoV spike models generated throughout this study, both structural and protein contact map superimpositions were performed. Three representative structures [SARS2 crystal structure (Figure 1A), SARS2 crystal model (Figure 1B), SARS2 sequence model (Figure 1C)] were used for superimposition. For model-crystal superimpositions, only spike monomers were considered. We found that RMSD values between crystal model:crystal [RMSD 1.78] (Figure 1D) and sequence model:crystal model [RMSD 1.77] (Figure 1E) were relatively close, which would imply that both generated models are structurally similar. In addition, the sequence model:crystal superimposition [RMSD 1.19] (Figure 1F) was below 1.5 Å which in-turn was considered adequate for further analyses (26). Furthermore, we observed that protein contact map superimposition between crystal model:crystal (Figure 1G), sequence model:crystal model (Figure 1H), and sequence model:crystal (Figure 1I) were above common contact 70%, which demonstrates the high contact similarity between the superimposed structures. Taken together, we believe that these results indicate that the generated models can be used for further downstream analyses.

### CoV Spike Models Differ Based on Phylogenetic Cluster and Lineage

**Figure 2:**
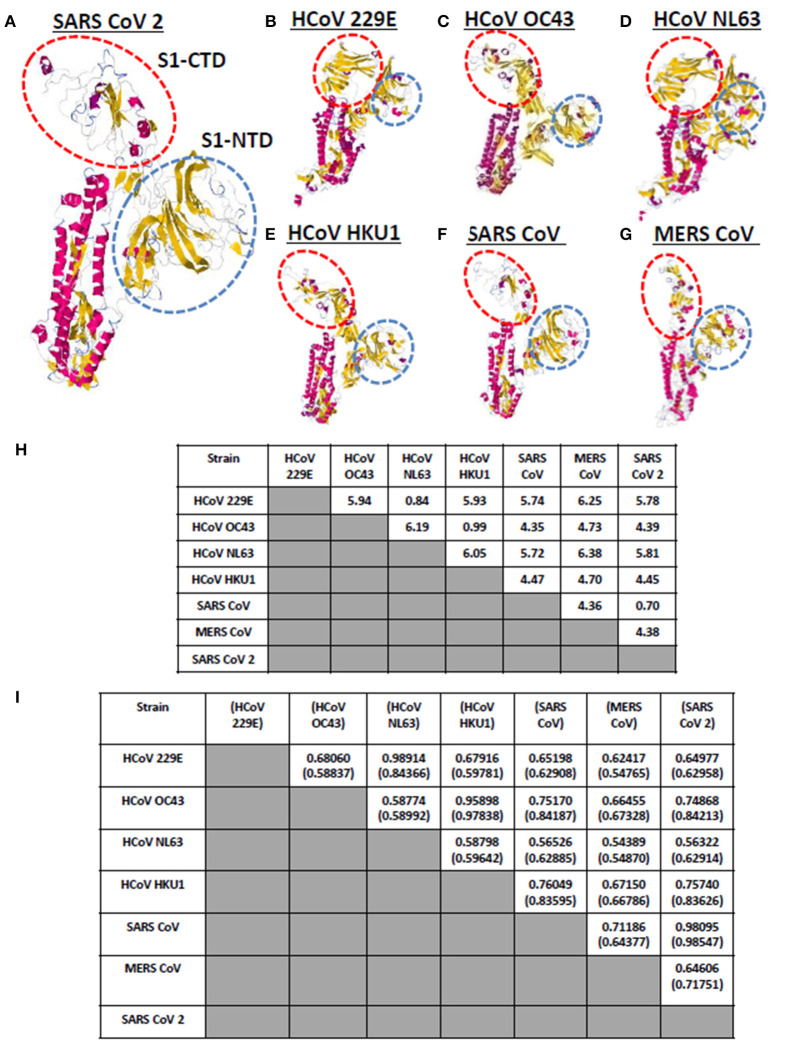
Whole protein structural comparison of the various monomeric human-infecting coronavirus spike protein models. Representative spike protein models of **(A)** SARS CoV 2, **(B)** HCoV 229E, **(C)** HCoV OC43, **(D)** HCoV NL63, **(E)** HCoV HKU1, **(F)** SARS CoV 1, and **(G)** MERS CoV are shown. S1-CTD (red dashed circle) and S1-NTD (blue dashed circle) are indicated. **(H)** RMSD and **(I)** Tm scores of superimposed spike models are tabulated. Tm scores normalized to a spike model is distinguished by having or not having a parenthesis.

Currently, there are seven known human-infecting CoV (1) and the spike protein for each CoV has been thoroughly studied (9, 10). CoV spike proteins are divided into two functionally distinct subunits (S1 and S2 subunits), wherein, the S1 subunit is further distinguished by four distinct domains (NTD comprised of domain A; CTD comprised of domains B, C, D) serving as receptor binding domains highlighting the importance of these domains in viral pathogenicity (9, 14, 27). To visualize and compare the CoV spike proteins, each human-infecting CoV spike model was generated and both visual observation and structural comparison mainly focused on both S1-CTD and S1-NTD. As seen in Figures 2A–G, through visual observation, a prominent structural difference between the spike models is the S1-CTD orientation (indicated in red dashed lines), whereas, S1-NTD orientation generally looked the same (indicated in blue dashed lines). More specifically, we were able to identify three possible patterns of spike S1-CTD orientation: (1) 229E and NL63; (2) OC43, HKU1, SARS1, and SARS2; and (3) MERS. Among the human-infecting CoVs, two strains (229E and NL63) belong to the alpha-CoV phylogenetic cluster, whereas, the remaining five strains belong to the beta-CoV phylogenetic cluster which can be further divided into the A (OC43 and HKU1), B (SARS1 and SARS2), and C (MERS) lineages (2–4, 6, 8, 28, 29). In this regard, we hypothesize that the similarities in S1-CTD orientation among the spike models is a possible structural feature associated to both the CoV phylogenetic cluster and lineage.

To further compare the CoV spike models, both RMSD value and Tm scores were determined. RMSD values measure similarity between two superimposed atomic coordinates, whereas, Tm scores measure the similarity between protein structures without relying on protein size (20, 30). Both measurements are used to establish structural similarities between two superimposed proteins (30). In this regard, we observed that superimposed CoV spike models that have RMSD <1.0 (Figure 2H) and Tm score > 0.95 (Figure 2I) either belong to the same phylogenetic cluster (229E and NL63) or lineage (OC43 and HKU1, SARS1 and SARS2, MERS). This would further imply that spike models in the same phylogenetic cluster or lineage generally may have similar protein structure as well. Noticeably, NL63 model normalized to the 229E model measured Tm score 0.84366 which is lower compared to other Tm scores measured from other CoVs spike models within the same phylogenetic cluster and lineage. This may suggest that there is some structural difference between these two spike models, which we suspect is related to viral evolution of NL63 from 229E (31).

It is worth mentioning that although all human-infecting CoVs are in the same protein structural fold (Tm score > 0.50) (32), among the superimposed spike models, we found certain beta-CoV strains that belong to separate lineages (OC43 and SARS1; OC43 and SARS2; HKU1 and SARS1; HKU1 and SARS2) have Tm score > 0.70 which (asides from being consistent to belonging to the same CoV genera) may likewise insinuate some degree of structural similarity albeit to a lesser extent compared to those in the same lineage (Tm score > 0.95). Additionally, in possible future works, it would be interesting to determine specific conformational features, establish known conformations and structural domains of S1-CTD, and elucidate domain classification among the seven human-infecting CoV spike protein.

### SARS1 and SARS2 Spike Models Are Structurally Similar

**Figure 3:**
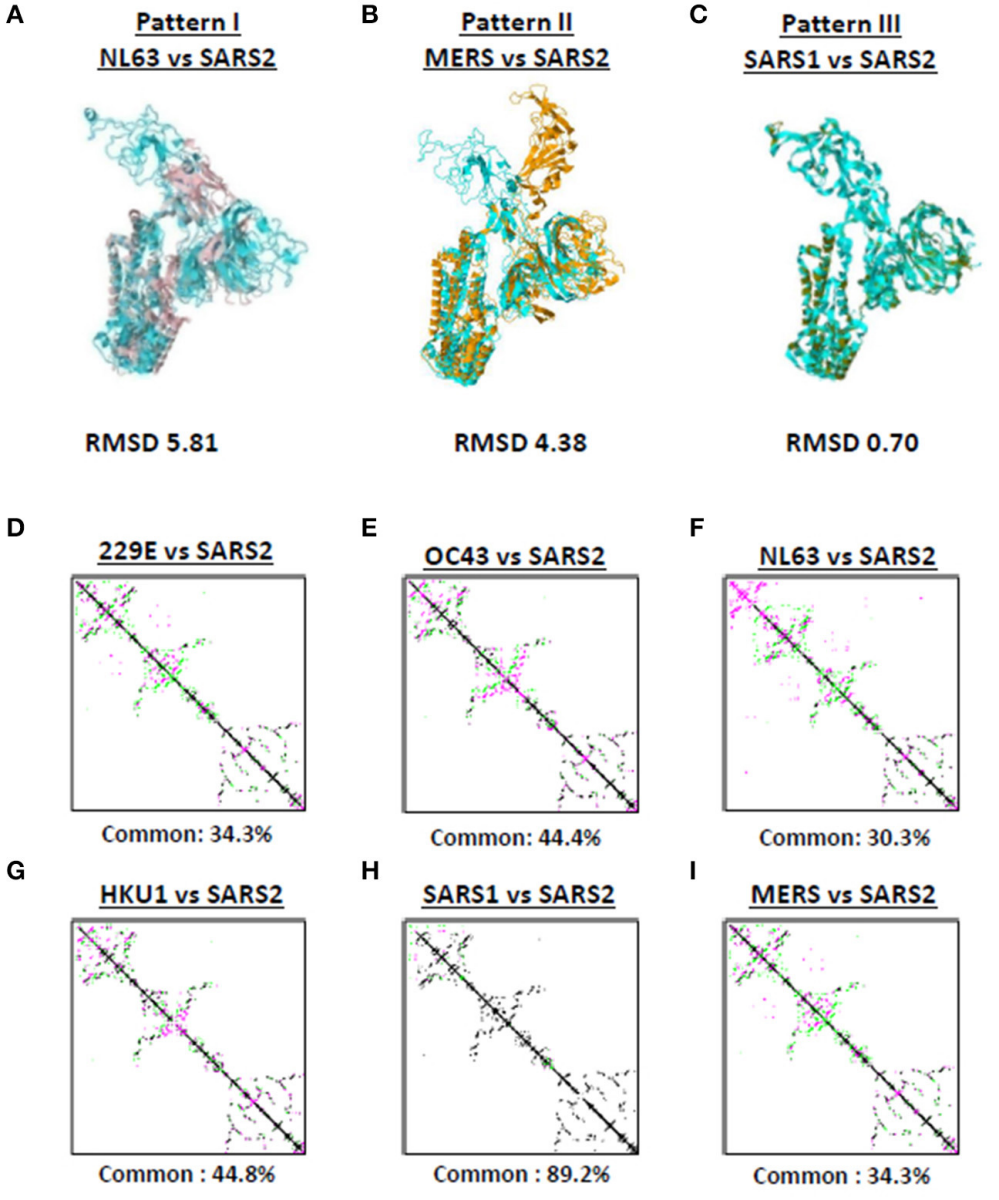
Putative structural patterns among the monomeric human-infecting conronavirus spike protein models. Representative **(A)** Pattern I based on superimposition of NL63 and SARS2 models, **(B)** Pattern II based on superimposition of MERS and SARS2 models, and **(C)** Pattern III based on superimposition of SARS1 and SARS2 models are shown. RMSD scores of the superimposed protein structures are indicated below. Contact maps of **(D)** 229E and SARS2, **(E)** OC43 and SARS2, **(F)** NL63 and SARS2, **(G)** HKU1 and SARS2, **(H)** SARS1 and SARS2, and **(I)** MERS and SARS2 models are shown. Contacts present in both protein structures (black) and present in one of the protein structures [either pink (first protein structure uploaded) or green (second protein structure uploaded: SARS2)] are indicated. Common contact of the protein structures being compared are labeled below.

Among the seven human-infecting CoVs, only SARS2 resulted to a pandemic (18, 19) which may suggest that the overall SARS2 spike protein differs from the other human-infecting CoVs. To structurally differentiate SARS2 and other human-infecting CoV spike models, model superimposition was performed. As a follow-up from our earlier results (Figure 1), we utilized representative spike models (NL63, MERS, SARS1) for superimposition against SARS2 since these models putatively share different S1-CTD orientation (based on visual observation) and have both RMSD <1.0 and Tm score > 0.95 among spike models within the same phylogenetic cluster and lineage. For purposes of this study, we classified distinct S1-CTD orientations as patterns and, likewise, established which among the spike protein models share the same S1-CTD orientation, whereby, spike protein models with the same S1-CTD orientation would be classified into one pattern. In this regard, we observed three distinct S1-CTD orientations which we classified into three patterns among the superimposed spike models: (1) Pattern I (NL63 and SARS2 superimposition; Figure 3A); (2) Pattern II (MERS and SARS2 superimposition; Figure 3B), and (3) Pattern III (SARS1 and SARS2 superimposition; Figure 3C). This is consistent with our earlier observations (Figures 2A–G) which would further suggest that spike models within the same phylogenetic cluster and lineage share the same spike S1-CTD model orientation. In this regard, based on Figures 2A–G, we think that 229E follows a Pattern I orientation while both OC43 and HKU1 follows a Pattern III orientation.

To further differentiate SARS2 and other human-infecting CoV spike models, contact map overlap (CMO) analyses were done. Contact maps provide information with regards to the pairwise spatial and functional relationship of residues in a given protein and, likewise, unifies certain aspects of protein folding and structure prediction which in-turn allows protein reconstruction (33, 34). We found that among the CoV spike models compared to SARS2 model (Figures 3D–I), only SARS1 has more common contact (89.2%) with SARS2 (Figure 3H) which in-turn would indicate that SARS1 and SARS2 spike models have high structural similarity compared to SARS2 and other human-infecting CoV spike models (common contact <50%). SARS1 and SARS2 viral genomes have ~80% nucleotide identity (35, 36), whereas, SARS1 and SARS2 spike proteins have a 75–81% nucleotide similarity (37). In this regard, asides from belonging to the same lineage (29), we correlated the high common contact between SARS1 and SARS2 spike models to high nucleotide similarity.

Considering the results at this point, it is worth mentioning that RMSD values, Tm score, and CMO analyses were all based on superimposition of full-length CoV spike protein models. However, since spike S1-CTD model orientation varied while spike S1-NTD model orientation seem to be the same (Figures 2A–G, 3A–C), structural comparison focusing only on both S1-CTD and S1-NTD is merited.

### SARS2 Spike S1-CTD and S1-NTD Models Have Some Degree of Similarity Among Beta-CoV Spike Models

**Figure 4:**
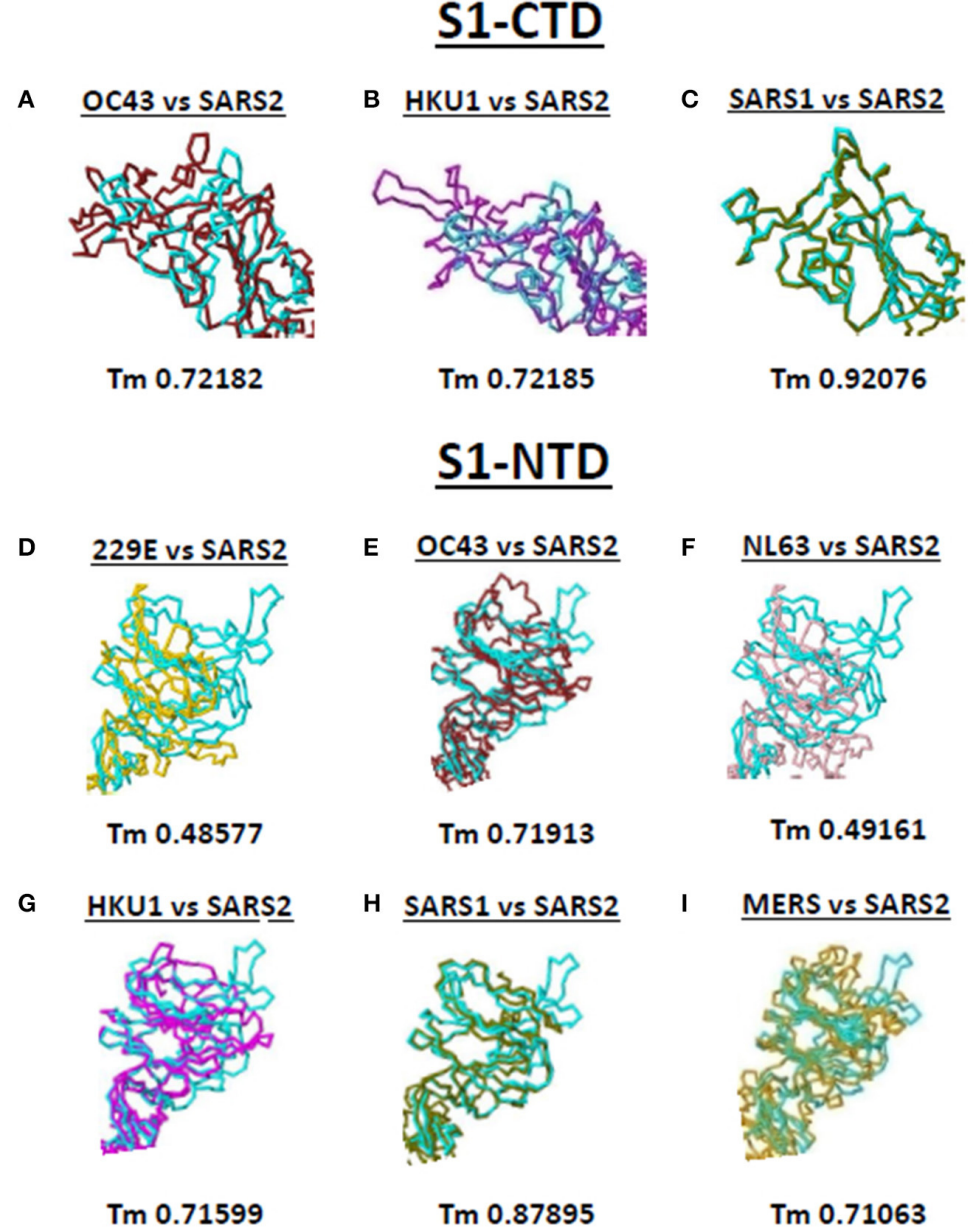
Structural comparison of S1-CTD and S1-NTD between the various human-infecting coronaviruses and SARS CoV 2 spike models. Spike S1-CTD comparison between superimposed **(A)** OC43 and SARS2, **(B)** HKU1 and SARS2, and **(C)** SARS1 and SARS2 models are shown. Spike S1-NTD comparison between superimposed **(D)** 229E and SARS2, **(E)** OC43 and SARS2, **(F)** NL63 and SARS2, **(G)** HKU1 and SARS2, **(H)** SARS1 and SARS2, and **(I)** MERS and SARS2 models are presented. Tm scores normalized to the SARS2 model are labeled below all superimposed structures. 229E (gold), OC43 (brown), NL63 (pink), HKU1 (magenta), SARS1 (olive), MERS (orange), and SARS2 (cyan) are indicated.

Both S1-CTD and S1-NTD are major domains located in the globular S1 subunit of CoV spike proteins that have been associated to receptor recognition (9, 10). Considering the spike S1-CTD orientation varied while the spike S1-NTD orientation were similar among the human-infecting CoVs, we compared the SARS2 spike S1-CTD and S1-NTD models from selected human-infecting CoV spike models through model superimposition. Subsequently, visual observation of the superimposed structure was performed and, for confirmation, Tm score normalized to either the SARS2 spike S1-CTD or S1-NTD model were likewise measured. For S1-CTD model superimposition, only human-infecting CoV spike models following Pattern III orientation were superimposed to the SARS2 spike S1-CTD model. For S1-NTD model superimposition, all spike models were used since all S1-NTD orientation seemed to be the same (Figures 2A–G). Based on visual observation, we observed that both SARS2 spike S1-CTD (Figures 4A–C, upper panels) and S1-NTD (Figures 4D–I, lower panels) model superimpositions showed few structural overlaps compared to other human-infecting CoV spike models, whereas, SARS2 spike S1-CTD model seems to suggest higher structural overlap with SARS1 spike model (Figure 4C, upper panel). Similarly, Tm score measurements of either SARS2 spike S1-CTD (Figures 4A–C, lower panels) or S1-NTD (Figures 4D–I, lower panels) model superimposition showed a Tm score > 0.70 except for 229E (Figure 4D) and NL63 (Figure 4F).

Taken together, we postulate that these results would insinuate that: (1) both SARS1 spike S1-CTD and S1-NTD have higher structural similarity (Tm > 0.90) with SARS2 spike S1-CTD and S1-NTD, respectively; (2) OC43 and HKU1 spike S1-CTD and S1-NTD have some degree of similarity (Tm > 0.70) with SARS2 spike S1-CTD and S1-NTD, respectively; (3) MERS spike S1-NTD may likewise have some degree of similarity (Tm > 0.70) with SARS2 spike S1-NTD; and (4) 229E and NL63 spike S1-NTD are not structurally similar (Tm <0.50) with SARS2 spike S1-NTD. We likewise suspect that this is correlated to whether the human-infecting CoV spike model belongs to the same phylogenetic cluster and lineage consistent with our earlier results.

## Discussion

SARS2 is the causative agent of coronavirus disease 2019 (COVID-19) pandemic (19). Interestingly, pre-existing SARS2 immunity has been observed among certain unexposed individuals in the general population (38, 39). Moreover, it was speculated that SARS2-specific T cells among unexposed individuals is attributable to memory T cells exposed to common cold CoVs (39) which in-turn would suggest the possible occurrence of immune cross-reactivity. By definition, cross-reactivity refers to immune responses that have non-specific targeting against a particular antigen ascribable to the flexible interaction between both B- and T-cell receptors and antigens (40). Considering all CoV infections start with spike protein binding, thereby, making it the first antigen recognized by the immune response (9, 10, 41), structural similarities between the spike proteins of human-infecting CoVs may play an important role in stimulating pre-existing SARS2 immunity. Throughout this study, we attempted to establish the putative structural differences and similarities among the seven known human-infecting CoVs spike protein conformations.

Epitopes serve as antigenic determinants that are normally found along the regions of an antigen that are recognized by B- and T-cells and can be classified as either sequential or conformational, whereby, sequential epitopes (continuous or linear amino acid stretch) do not rely on protein conformation while conformational epitopes (discontinuous amino acid stretch) rely on protein folding and conformation (42–44). In addition, conformational epitopes make up ~90% of total antigen:antibody complexes (45) which would emphasize the importance of conformational epitopes. On the other hand, complimentary determinants (paratopes) within the antibody variable region recognize and interact with epitopes, wherein, this particular interaction goes beyond amino acid sequence recognition but rather is at the level of epitope:paratope steric complementarity and ionic charge (40). Considering every antibody paratope could interact with multiple antigen epitopes, this could lead to polyclonal immune response which is a fundamental principle of cross-reactivity (40, 46). Thus, this would mean that antibody binding to conformational epitopes could potentially lead to cross-reactivity. Considering the CoV S glycoprotein is the primary target for neutralizing antibody (47), we assume that any possible structural similarities between CoV spike proteins would likewise mean putatively comparable conformational epitopes. Earlier works have shown that immune cross-reactivity (and some cases of cross-neutralization) among human-infecting CoVs has been observed in the following human-infecting CoVs: between SARS1 and SARS2 (48–50); between SARS1 and NL63 (51); between SARS1 and 229E (51, 52); between SARS1 and OC43 (51–53); between SARS1 and MERS (54); and between NL63 and 229E (55). In this regard and considering our results, we hypothesize that some degree of structural similarity (Tm > 0.70) between SARS2 and other human-infecting CoVs spike S1-CTD and S1-NTD may suggest the possibility of cross-reactivity, whereby, potential neutralizing antibodies that recognize conformational epitopes along the S1-CTD and S1-NTD of human-infecting CoVs spike protein could likewise recognize conformational epitopes along the SARS2 spike S1-CTD and S1-NTD. In fact, consistent with our proposed hypothesis, earlier works have shown that pre-existing T cells recognizing SARS2 can be detected in a significant portion of the global human population (38, 39, 56) possibly attributable to humans being exposed to at least one form of human-infecting CoV (57). Thus, we believe that this would further support the possibility of having pre-existing immunity against SARS2 via cross-reactive immune response from other human-infecting CoVs with at least some degree of structural similarity (particularly in S1-CTD and S1-NTD).

It is worth mentioning that levels of neutralizing antibody response between human-infecting CoVs may likewise vary as previously observed (52), wherein, SARS1 and OC43 cross-reactive immune response was found to be higher compared to SARS1 and 229E cross-reactive immune response. Considering the results we obtained in this study, we speculate that varying immune cross-reactivity among human-infecting CoV spike protein might be ascribable to whether one or both S1-CTD and S1-NTD have higher structural similarity which in-turn may be influenced by both CoV phylogenetic cluster and lineage. Moreover, since immune responses (both humoral and cellular) to CoV diminishes at a certain time which in-turn allow for future re-infection (58–60), we likewise suspect that this may impact immune cross-reactivity of SARS2 and other human-infecting CoVs which consequently may affect the severity of SARS2 infection. To speculate on the impact, patients with a relatively recent CoV infection (not SARS2) may develop a less severe form of COVID-19 while patients infected by another human-infecting CoV more than a year ago may result into a more severe form of COVID-19 with both scenarios being affected by the presence or absence of cross-reactive immune response from a prior human-infecting CoV contagion with some degree of structural similarity to one or both SARS2 S1-CTD and S1-NTD. We emphasize that these are speculations and would ultimately require both laboratory and clinical experimentation to prove. Similarly, we would like to highlight that the entire study is performed with predicted monomeric protein conformations, however, in cells a trimer of spike protein usually attaches to the host receptor (depending on the human-infecting CoV strain). In this regard, our results and interpretation to these results may differ in a CoV infection scenario.

In summary, we putatively established the differences and similarities in the structural conformation of the spike protein among human-infecting CoVs. In particular, we postulate on the following: (1) structural orientation of S1-CTD is a possible structural feature associated to both the CoV phylogenetic cluster and lineage; (2) spike models in the same phylogenetic cluster or lineage could potentially have similar protein structure; (3) there are potentially three distinct S1-CTD orientation among the human-infecting CoVs; and (4) human-infecting CoVs in the same phylogenetic cluster possibly have similar S1-CTD and S1-NTD. Overall, we propose that the SARS2 spike S1-CTD follows a Pattern III orientation which has a higher degree of similarity with SARS1 and some degree of similarity with both OC43 and HKU1 which coincidentally are in the same phylogenetic cluster and lineage, whereas, the SARS2 spike S1-NTD has some degree of similarity among human-infecting CoVs that are either in the same phylogenetic cluster or lineage.

## Data Availability Statement

The raw data supporting the conclusions of this article will be made available by the authors, without undue reservation.

## Author Contributions

Both authors were involved in formulating the idea, performing the structural analyses, and writing the manuscript.

## Conflict of Interest

The authors declare that the research was conducted in the absence of any commercial or financial relationships that could be construed as a potential conflict of interest.
